# Healing sequelae following tooth extraction and dental implant placement in an aged, ovariectomy model

**DOI:** 10.1093/jbmrpl/ziae113

**Published:** 2024-08-31

**Authors:** Jessica M Latimer, Shogo Maekawa, Takahiko Shiba, Tobias Fretwurst, Michael Chen, Lena Larsson, James V Sugai, Paul Kostenuik, Bruce Mitlak, Beate Lanske, William V Giannobile

**Affiliations:** Department of Oral Medicine, Infection, and Immunity, Harvard School of Dental Medicine, Boston, MA 02115, United States; Department of Periodontology, Graduate School of Medical and Dental Sciences, Tokyo Medical and Dental University, Tokyo 113-8510, Japan; Department of Oral Medicine, Infection, and Immunity, Harvard School of Dental Medicine, Boston, MA 02115, United States; Department of Periodontology, Graduate School of Medical and Dental Sciences, Tokyo Medical and Dental University, Tokyo 113-8510, Japan; Department of Oral Medicine, Infection, and Immunity, Harvard School of Dental Medicine, Boston, MA 02115, United States; Department of Oral and Craniomaxillofacial Surgery/Translational Implantology, Faculty of Medicine, Medical Center, University of Freiburg, Freiburg 79106, Germany; Department of Oral Medicine, Infection, and Immunity, Harvard School of Dental Medicine, Boston, MA 02115, United States; Department of Oral Biochemistry, Institute of Odontology, Sahlgrenska Academy, University of Gothenburg, Gothenburg 413 90, Sweden; Department of Oral Medicine, Infection, and Immunity, Harvard School of Dental Medicine, Boston, MA 02115, United States; Department of Periodontics & Oral Medicine, University of Michigan School of Dentistry, Ann Arbor, MI 48109, United States; Department of Periodontics & Oral Medicine, University of Michigan School of Dentistry, Ann Arbor, MI 48109, United States; Phylon Pharma Services, Thousand Oaks, CA 91320, United States; Radius Health Inc., Boston, MA 02210, United States; Radius Health Inc., Boston, MA 02210, United States; Department of Oral Medicine, Infection, and Immunity, Harvard School of Dental Medicine, Boston, MA 02115, United States

**Keywords:** bone regeneration, implant, tooth extraction, aging, osteoporosis

## Abstract

At present, a lack of consensus exists regarding the clinical impact of osteoporosis on alveolar bone metabolism during implant osseointegration. While limited preclinical and clinical evidence demonstrates a negative influence of osteoporosis on dental extraction socket healing, no preclinical studies offer data on the results of implant placement in 6-mo-old, ovariectomized (OVX) Sprague–Dawley rats. This study aimed to investigate the outcomes of dental tooth extraction socket healing and implant placement in a rodent model of osteoporosis following daily vehicle (VEH) or abaloparatide (ABL) administration. Micro-CT and histologic analysis demonstrated signs of delayed wound healing, consistent with alveolar osteitis in extraction sockets following 42 d of healing in both the VEH and ABL groups. In a semiquantitative histological analysis, the OVX-ABL group demonstrated a tendency for improved socket regeneration with a 3-fold greater rate for moderate socket healing when compared to the OVX-VEH group (43% vs 14%), however, this finding was not statistically significant (*p*=.11). No significant differences were observed between vehicle and test groups in terms of implant outcomes (BMD and bone volume/total volume) at 14- and 21-d post-implant placement. Abaloparatide (ABL) significantly increased BMD of the femoral shaft and intact maxillary alveolar bone sites in OVX animals, demonstrating the therapeutic potential for oral hard tissue regeneration. The present model involving estrogen-deficiency-induced bone loss demonstrated an impaired healing response to dental extraction and implant installation.

## Introduction

Osteoporosis is a systemic disease characterized by loss of bone mass and microarchitectural deterioration, resulting in increased skeletal fragility and susceptibility to life-threatening fractures.^1^ Approximately 10 million Americans are affected by osteoporosis; one in two women and one in four men, over the age of 50 will experience a fragility fracture due to osteoporosis.^2^ The influence of osteoporosis on oral bone metabolism remains inconclusive, though there are studies that link an increase in alveolar bone loss and tooth loss in periodontitis patients to low systemic BMD.^3^ Periodontitis is a chronic inflammatory disease in which biofilm triggers host dysbiosis that results in loss of alveolar bone.^4-8^ Interestingly, several of the systemic health factors that are associated with osteoporosis, such as age, genetics, smoking, nutritional deficiencies, and hormonal changes (ie, menopause), are also linked to periodontal disease pathogenesis.^3^

Emerging evidence indicates a complex interplay of aging and oral inflammation in the alveolar bone metabolism of patients with osteoporosis.^3^ The risk of osteoporosis is strongly related to advanced age and aging has been associated with physiologic deterioration, genomic and epigenomic alterations, and impaired cellular functions.^9-11^ As a population with a higher susceptibility to tooth loss, the wound-healing process of alveolar bone in response to dental surgical procedures of the alveolus is of clinical consequence for patients affected by osteoporosis.^3^ Despite the age-related functional decline in systemic tissue repair and homeostasis, biological age alone has not been shown to influence implant osseointegration or survival clinically.^12^ Current evidence also fails to demonstrate higher rates of implant failure in individuals with osteoporosis.^13^^,^^14^ Nonetheless, osteoporosis has been associated with increased susceptibility to peri-implant bone loss^14^ and is an established risk indicator for peri-implant diseases.^15^ Pharmacological therapies for osteoporosis such as bisphosphonates or anti-RANKL monoclonal neutralizing antibody (denosumab) introduce additional considerations in surgical dental treatment due to the potential risk for developing medication-related osteonecrosis of the jaw (ONJ).^3^^,^^16^

One class of osteoporosis drugs with potential for use in oral regeneration comprises the parathyroid hormone 1 receptor (PTH1R) agonists. Systemic administration of teriparatide, the biologically active, amino-terminal 34-amino-acid fragment of PTH (1-84), has been demonstrated to improve bone regeneration of osseous defects in alveolar bone significantly and to enhance clinical parameters of periodontal health.^17^ Abaloparatide (ABL), a 34-amino-acid analog of human PTHrP, selectively activates PTH1R signaling to promote anabolic activity. In the treatment of osteoporosis, ABL promotes greater bone density gains with less bone resorption when compared to teriparatide.^18^ In vitro data indicate that certain PTHrP analogs can distinguish between two high-affinity PTH1R conformations, R0 and RG and that ABL selectively binds to the RG PTH1R configuration, inducing a transient signaling response that favors bone formation with lesser increases in bone resorption and blood calcium compared with R0 binding.^19^ This mechanism may account for the superior bone anabolic effects of ABL as compared to teriparatide in clinical trials, suggesting scientific and clinical value in determining alveolar bone responses to ABL.

While dental implants are a highly efficacious treatment modality for tooth loss, implant therapy may present challenges in the treatment of osteoporosis patients due to concerns regarding bone quantity (limited mass and volume), quality (inferior microarchitectural and mechanical properties), or peri-implant bone healing (impaired bone metabolism affecting extraction socket healing or implant osseointegration).^20-22^ Despite the possible deleterious effects of osteoporosis on dental implant surgery, there are at present no preclinical studies having studied implant installation following tooth extraction in an osteoporosis in vivo rodent model using 6-mo-old ovariectomized animals. In the existing preclinical literature, favorable regenerative capacity for extraction socket healing and implant osseointegration has been established in young (3-5 wk), male rats.^23-25^ The present study aimed to analyze (1) alveolar bone healing following maxillary tooth extraction, implant osseointegration, and peri-implant regeneration in a rat model of postmenopausal osteoporosis and (2) to investigate the effects of ABL, a PTHR1 agonist that is FDA-approved for the treatment of osteoporosis, on alveolar bone regeneration.

## Materials and Methods

### Preclinical model of alveolar bone defect from tooth extraction in post-menopausal osteoporosis

All animal procedures were performed with approval from the Harvard Institutional Animal Care and Use Committee according to the ARRIVE guidelines for preclinical studies (IACUC Protocol ID #IS00003379). Female Sprague–Dawley rats were ovariectomized (OVX) or sham-operated, respectively, at 16 wk of age and housed for 8 wk by a commercial vendor (Charles River Laboratories) to allow for a period of bone depletion. At 24 wk of age, the animals (4 animals/cage) underwent a 7-d acclimation period upon arrival at the animal housing facility at the Harvard Center for Comparative Medicine. The total number of animals was 64. Twenty-four animals (Sham-VEH; *n* = 8, OVX-VEH; *n* = 8, and OVX-ABL; *n* = 8) received unilateral extraction of the right maxillary first molar (M1) and were sacrificed at 6 wk post-extraction, when they were 7.5 mo old. Forty animals received bilateral M1 extraction followed by bilateral osseous defect creation and implant placement 6 wk post-extraction. Animals were sacrificed at 14 d (OVX-VEH; *n* = 9, OVX-ABL; *n* = 7) or 21 d (OVX-VEH; *n* = 13, OVX-ABL; *n* = 11) post-implant placement.

**Figure 1:**
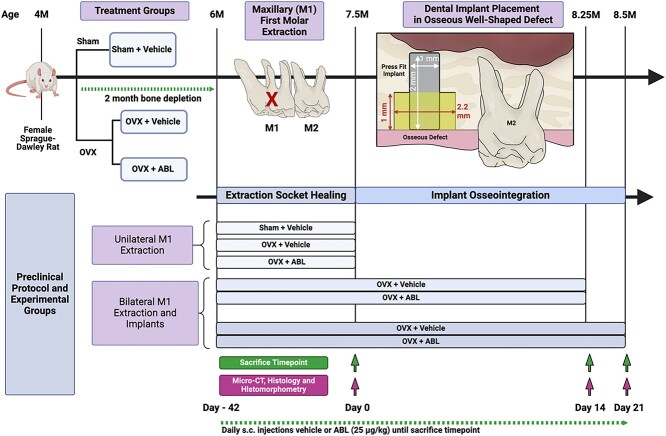
Study design. Overview of experimental design. Four-month-old female Sprague–Dawley rats underwent a sham operation or ovariectomy (OVX) surgery to induce estrogen-deficiency. Animals were aged for 2 mo to allow for bone depletion. At 6 mo of age, animals underwent: (1) unilateral extraction of the maxillary first molar (M1) and sacrifice after 42 d socket healing; or (2) bilateral extraction of the maxillary M1 and received press-fit dental implants placed in osseous well-shaped defects at 42 d, followed by biopsy harvest at 14- or 21-d post-implant placement.

One day before surgery, the animals received subcutaneous (s.c.) injections of carprofen analgesic (5 mg/kg). Under general anesthesia using isoflurane (4% induction, 1%-2% maintenance), the maxillary M1 was extracted unilaterally (extraction socket healing only groups) or bilaterally (implant placement groups) using an atraumatic technique. Immediately following tooth extraction, the root sockets were irrigated with 0.9% normal saline and daily s.c. injections of ABL (25 μg/kg) or vehicle (saline) were initiated. Treatment group was allocated randomly. After 6 wk of socket healing, the unilateral M1 extraction groups were sacrificed, and bilateral M1 extraction groups underwent surgery for bilateral implant placement. The surgical operators (J.L. and S.M.) performed all procedures masked to the treatment allocations. A 1 cm crestal incision was made along the healed, edentulous M1 ridge, and a full-thickness mucoperiosteal flap was elevated. Thereafter, an osteotomy with a standardized, well-shaped osseous defect measuring 2.2 mm in diameter in the coronal half and 0.95 mm in diameter in the apical half was created using a custom carbide step drill under copious irrigation with sterile saline as previously described.^26^ Surgical site preparation was followed by bilateral press-fitting installation of custom-fabricated, sterile, commercially pure, solid-cylinder titanium implants (1 mm diameter, 2 mm length) with a titanium sandblasted, large grit, acid-etched surface (Institut Straumann AG, Waldenburg, Switzerland). The flap edges were approximated to obtain primary closure with 8-0 absorbable polyglactin 910 (Flysorb©, Butterfly Italia, S.r.l, Cavenago di Brianza MB, Italy) sutures. The implants remained submerged and unloaded throughout the healing period. Following the extraction and implant procedures, multimodal pain control was performed with buprenorphine (1 mg/kg, s.c.) and carprofen (5 mg/kg, s.c.) immediately postoperatively, as well as with additional carprofen administration (5 mg/kg, s.c.) for 2 d. For animals receiving implants, oral administration of an antibiotic, enrofloxacin (10 mg/kg), in 0.1 mL glucose solution was performed 1 d before surgery and continued for 7 d postoperatively. A soft gel diet, nutritional supplement, and drinking water with ampicillin (268 μg/ml) were used for 7 d postoperatively. Daily monitoring was performed by the university veterinary staff and surgical team. Weight was measured in grams daily from the time of extraction until sacrifice to validate the effect of OVX treatment. OVX-treated animals demonstrated significantly higher body weights at the time of M1 extraction and 1, 2, 3, 4, 5, and 6 wk post-extraction (*p*<.0001) (Supplemental Figure S1). Increased body mass has been used as a proxy for the effect of estrogen depletion in OVX rats.^27^ An overview of the sample sizes, experimental groups, and surgical protocol is summarized in Figure 1.

### Maxillae harvest, micro-CT, histological preparation, and analysis

The animals were euthanized by carbon dioxide inhalation overdose at the designated sacrifice time points. Posterior maxillary specimens were harvested, dissected, and fixed in 10% neutral-buffered formalin for 2 d. The specimens were transferred to 70% alcohol and placed in 34 mm diameter specimen holders for micro-CT scanning (μCT100 Scanco Medical, Bassersdorf, Switzerland). The scan settings used were as follows: voxel size 18 μm, 90 kVp, 44 μA, 0.5 mm AL filter, and integration time 1000 ms. For the acquisition of the micro-CT images, all specimens were oriented along the sagittal plane. Scans were reconstructed and two- and three-dimensional images were generated for all specimens. Micro-CT analysis was performed following protocols described previously.^24^^,^^25^ In the unilateral extraction group, BMD was measured in the intact and extraction site using the mesial root socket as the region of interest (M1, 70 slices, threshold 184 HU). In the implant groups, titration of the halo effect was done and the newly formed bone volume (Bone Volume Fill, BVF), as well as the BMD, were calculated within the defect and around the entire implant. Evaluation of the femur samples was performed using the midshaft as the region of interest (bone volume (BV), total volume (TV), BMD, 100 slices, threshold 184 HU). Analysis was performed by an experienced, masked examiner (T.F.) using Scanco software (Scanco Medical AG, Brüttisellen, Switzerland).

Decalcified and undecalcified sections were prepared for histologic and histomorphometric analyses. Samples were decalcified in 10% EDTA for 3 wk, embedded in paraffin, and 5 μm sections were obtained. A sagittal section of the maxilla was made to analyze extraction sockets and interradicular bone. The sections were stained with toluidine blue and basic fuchsin for histological analysis. Undecalcified sections were dehydrated in step gradients of alcohol, infiltrated, and embedded in MMA. Cross-sectional sections of ~50-μm thickness were cut along each implant’s long axis using a diamond saw at the central portion of each implant (Isomet Low-Speed Saw, BUEHLER, United States). The sections were attached to plastic tissue slides, ground down to less than 20 μm with an Ecomet 300 Pro Grinder-Polisher (Buehler, United States), and polished. The sections were then stained with toluidine blue and basic fuchsin according to previous protocols with slight modifications. Briefly, sections were placed in 0.1% formic acid for 5 min, quickly rinsed in distilled water (dH_2_O), dipped into 70% ethanol for 15 min, followed by staining with 1% toluidine blue for 5 min. After rinsing with dH_2_O, sections were dipped in 70% ethanol for 1 min, and 1% basic fuchsin for 1 min, rinsed again with dH_2_O, dehydrated in a gradient of alcohol, and air dried. Microscopic images were captured with a Keyence BZ-X700E microscope (Keyence Corp. of America, Itaca, IL, United States). A semiquantitative analysis was performed for the coded specimens by a single experienced examiner (T.F.).

### Statistical analyses

Analysis of micro-CT data were performed by one-way ANOVA followed by Tukey’s multiple comparison test to compare pairwise group differences using GraphPad Prism (version 10.0.2 for Mac, GraphPad Software, Boston, MA, United States). The data were presented as the mean ± standard deviation. A value of *p*<.05 was considered statistically significant. The minimum sample size to attain statistical significance of *p*<.05 with 80% power was 4 animals per group based on results for peri-implant BMD from a previous study.^28^

## Results

The final sample sizes were 23 animals (sham-VEH; *n* = 8, OVX-VEH; *n* = 7, and OVX-ABL; *n* = 8) in the unilateral extraction cohort and 39 animals with 57 implants in the bilateral implant cohort. Sixteen animals were sacrificed at 14 d (OVX-VEH; *n* = 9 with 12 implants, OVX-ABL; *n* = 7 with 8 implants) post-implant placement and 23 animals were sacrificed at 21 d (OVX-VEH; *n* = 12 with 20 implants, OVX-ABL; *n* = 11 with 17 implants) post-implant placement. Due to surgical complications, early implant loss, or artifacts with histological processing, the total number of specimens was reduced to 7-8 animals per group in the unilateral extraction cohort, 7-9 animals (8-12 implants, 37.5% overall) in the D14 implant group, and 11-12 animals (17-20 implants, 22.9% overall) in the D21 implant group. No statistically significant relationship to the test or control treatment in the excluded specimens was noted.

### Clinical findings

**Figure 2:**
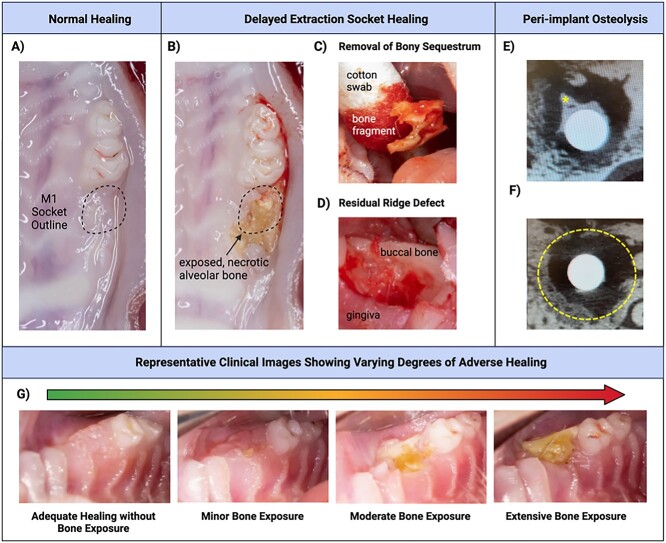
Clinical findings. (A) Example of normal alveolar bone ridge healing without residual root tips, ridge defect, or bone exposure apparent clinically. (B) Adverse healing outcome involving necrotic, exposed bone. (C) Removal of exfoliated bone sequestrum. (D) Example of ridge defect with significant concavity in extraction site precluding implant placement. (E-F) Representative images of partial peri-implant healing with some bone formation on the implant surface (asterisk) amidst poorly defined lucent, mixed, or sclerotic lesion, sequestrum, periosteal proliferation and/or destruction of adjacent structures (radiological signs of an osteonecrosis). Dashed line shows peri-implant osseous well-defect border. (G) Representative clinical intraoral images showing varying degrees of osteonecrosis, from no visible bone exposure to mild, moderate, and extensive bone exposure.

Following tooth extraction, many animals exhibited a delayed wound healing process, with some showing alveolar osteitis characterized by large areas of necrotic, exposed bone. The extent of osteonecrosis-like lesions in M1 sites was clinically evaluated in each animal before implant placement. Adequate healing was considered as a clinical appearance of intact soft tissue without bone exposure. Upon surgical entry, a lack of bone formation and sclerotic bone in the former extraction socket alveolus were observed in some animals. Sockets with adverse healing outcomes were characterized by minor, moderate, or extensive regions of exposed or necrotic bone. The most severe cases of poor healing were accompanied by osteolysis extending to the maxillary sinus floor and adjacent teeth. Exfoliated bone sequestra were observed in the most superficial aspect of the mucosal tissue. Underlying these areas of necrotic bone, a residual ridge defect indicating compromised extraction socket healing was often detected. These clinical findings are summarized in Figure 2. Extraction sites with focal regions of exposed bone or fistulae that extended to bone were considered mild exposure. Extraction sites with moderate exposure approximating the area of the original extraction socket to severe bone exposure extending to adjacent structures and/or significant ridge defects did not receive implants. Bone samples obtained from the extraction sites were assessed by aerobic and anaerobic cultures for microbial identification and susceptibility testing. Osteomyelitis or overt bacterial infection of the extraction socket was ruled out by the culture results which were negative for anaerobic growth; the panel of aerobic species included moderate growth for *Escherichia coli, Citrobacter*, and *Beta-hemolytic streptococci* while *Enterococcus gallinarum* growth was absent. A clinical veterinarian was consulted to confirm that the bacterial species identified was consistent with normal flora in the rat oral microbiome.^29^

### Micro-CT findings

**Figure 3:**
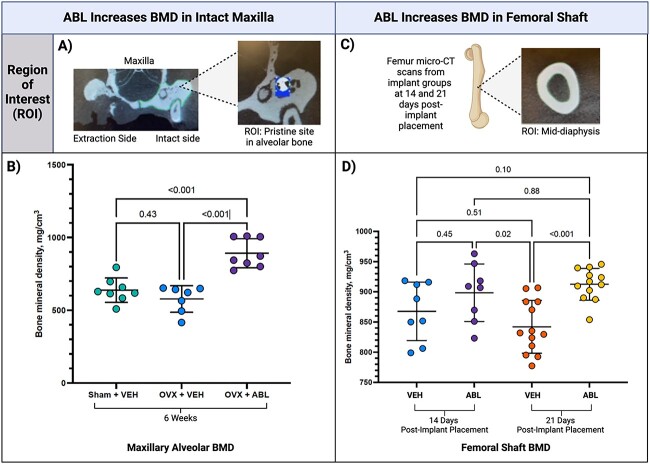
Micro CT results in the femoral shaft and intact maxilla. Micro-CT analysis was performed to assess BMD in (A) intact maxillary alveolar sites where (B) BMD was greatest in the ovariectomized (OVX) group receiving abaloparatide (ABL) (*p*<.001). Between the two groups receiving vehicle injection, there was a trend for decreased BMD in the OVX animals. BMD in the (C) femur was assessed using the center of the diaphyseal portion and (D) BMD was greatest at 21 d post-implant placement in the group receiving ABL compared to the control (*p*<.001).

In the animals that received unilateral extractions and VEH or ABL injection for 6 wk, no significant difference was found in the BMD of healed extraction sockets. However, a significant difference was detected in the intact alveolar ridge of the contralateral maxillary M1 sites. Maxillary alveolar BMD was greater in the OVX-ABL group (*p*<.001) compared to the sham-VEH and OVX-VEH groups (Figure 3A-B). In the sham-VEH and OVX-VEH animals in the unilateral extraction cohort, there was a trend for decreased maxillary alveolar BMD in the OVX animals vs sham controls.

**Figure 4:**
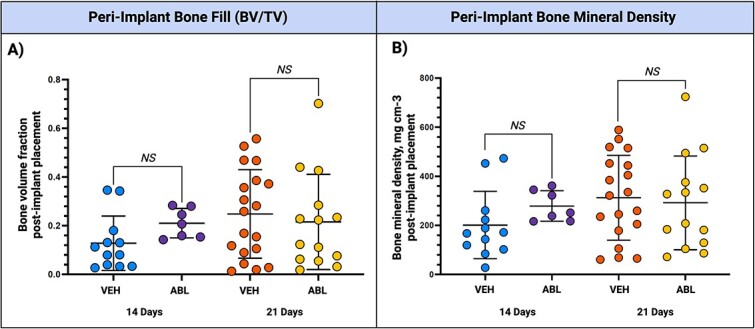
Micro CT results: peri-implant bone fill and peri-implant bone density. Micro-CT analysis was performed to assess (A) peri-implant bone fill (BV/TV) and (B) peri-implant BMD in the peri-implant osseous well-shaped defect. No significant differences were detected between groups for either outcome.

After 9 wk of treatment in the D21 implant group, femoral shaft BMD was significantly greater in the group receiving ABL compared to the group receiving VEH (*p*<.001) (Figure 3C-D). One implant in the D14 implant group and four implants in the D21 implant group were excluded from micro-CT analysis due to displacement or adjacent tooth remnants. No defect was completely healed. Among the D14 and D21 implant groups overall, 61.4% of the samples (*n* = 35) exhibited a poorly defined lucent, mixed, or sclerotic lesion, sequestrum, periosteal proliferation, and/or destruction of adjacent structures (radiological signs consistent with osteonecrosis). In some samples, a modest amount of new bone formation was observed directly on the implant surface. In two samples, bone fractures were present. The micro-CT analysis of peri-implant bone fill (BV/TV) and peri-implant BMD in the peri-implant osseous well-shaped defect showed no significant differences between the D14 and D21 implant groups (Figure 4).

### Histological findings

#### Extraction socket healing

**Figure 5:**
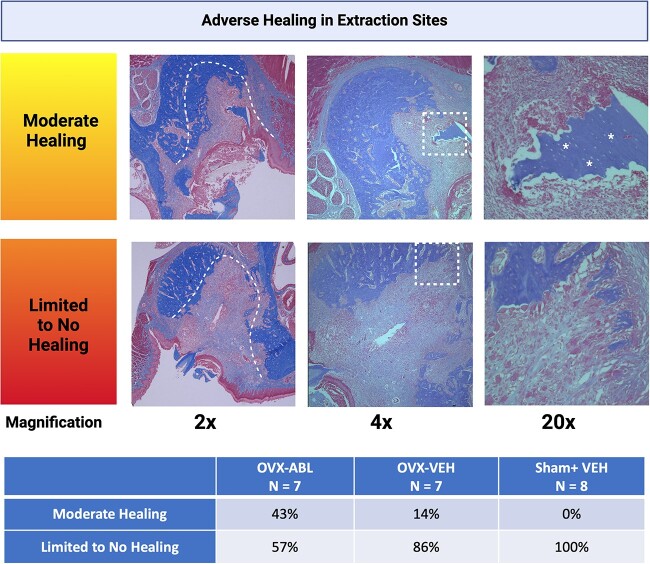
Histopathological spectrum of osteonecrosis in extraction sites. Histology of extraction sockets with moderate, limited, or no healing at 2×, 4×, and 20× magnification. In the 2× view, the dashed lines indicate the socket borders. In the 2× view of the sample with limited to no healing, the socket is filled entirely with connective tissue without signs of inflammatory infiltrates. The dashed, square boxes in the 4× view indicate the region of magnification in the accompanying 20× view. The arrows in the 4× view of the sample with limited healing indicate bone apposition characterized by thickening or sclerosis of the basal aspect of the maxilla. In the 20× views of the samples with moderate and limited healing, asterisks mark the isolated sequestra of necrotic bone filled with numerous empty osteocytic lacunae. A chi-squared test revealed no significant difference in the number of samples with moderate or limited to no healing between treatment groups. (*p* = .1151).

The extraction sockets of animals receiving VEH or ABL injection exhibited minimal new bone formation, but instead, a predominantly, cell-rich, and well-vascularized connective tissue without signs of inflammatory infiltrates was seen after 42 d of healing. Applying a semiquantitative histological analysis, 100% of the animals in the sham-VEH group showed limited to no socket healing, characterized by a lack of bone formation throughout the socket. The OVX-ABL group demonstrated a slight tendency for improved socket regeneration with a 3-fold greater rate for moderate socket healing, which consisted of any amount of socket fill, when compared to the OVX-VEH group (43% vs 14%), however, this finding was not statistically significant (*p* = .1151) (Figure 5). Isolated sequestra consisting of necrotic bone, as evidenced by empty osteocytic lacunae, were detected in numerous samples, and appeared to have exfoliated to the mucosal surface. Regions of bone apposition characterized by thickening or sclerosis of the basal aspect of the maxilla were also identified. The mucosal lining overlying the M1 site appeared intact in most samples.

#### Implant healing

In the bilateral extraction and implant groups, a significant number of samples exhibited histological evidence of peri-implant osteonecrosis at 14- and 21-d post-implant placement. The adverse healing was characterized by a disrupted mucosal lining, a high prevalence of empty osteocytic lacunae, the formation of bone sequestra, and new bone apposition at the basal aspect of the maxilla. The peri-implant defects displayed minimal new bone formation or a complete lack of osseous filling, along with a substantial area of osteolysis accompanied by the presence of connective tissue ingrowth surrounding the implant site. Some implants appeared to be dislocated.

## Discussion

To our knowledge, this study was the first to evaluate post-extraction implant placement in a model of estrogen-deficiency bone loss. In the present study, delayed extraction socket healing and a high incidence of alveolar osteitis were observed in the clinical, histological, and radiological qualitative assessments performed. These findings are in line with existing pre-clinical literature evaluating extraction socket healing in ovariectomized rats; a recent systematic review reported adverse effects of osteoporosis on alveolar socket repair, such as delayed healing and reduced volume of new bone formation, in 88% of 25 included studies.^30^ Of note, in the four studies that utilized Sprague–Dawley rats,^31-34^ none reported clinical findings of osteonecrosis following tooth extraction in the OVX groups receiving control treatments. However, these studies either used young animals or shorter periods of bone depletion inadequate to simulate the effects of osteoporosis in alveolar bone.^35^ Because bone formation peaks at 3 mo of age in Sprague–Dawley rats, simultaneous skeletal growth in younger animals may obscure any changes in alveolar bone related to OVX surgery.^35^

**Figure 6:**
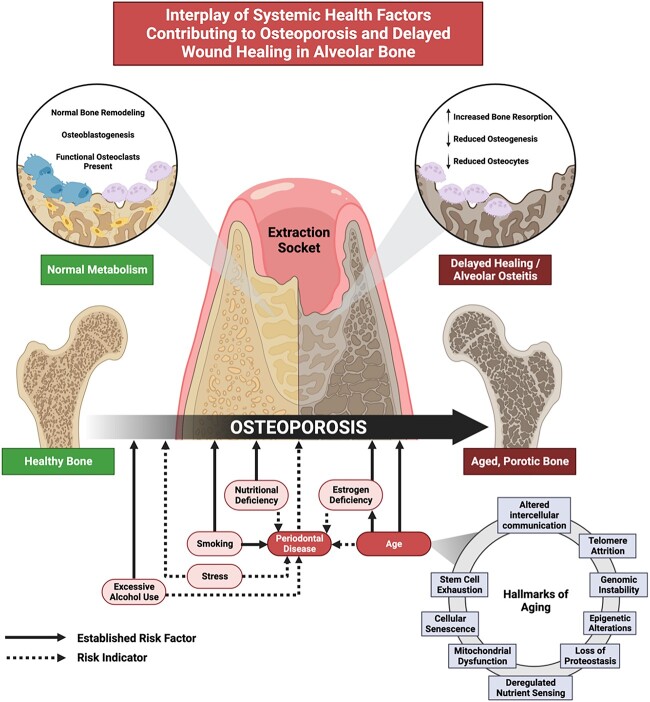
Proposed mechanistic overview of alveolar bone healing. A complex interplay of health factors related to oral and systemic bone disease may contribute to altered extraction socket healing. A proposed mechanistic diagram demonstrates some of the relationships between aging, periodontal disease or inflammation, and osteoporosis.

While no other studies assess implant placement in rodent models of osteoporosis, one study performed osteotomy preparation alone in 12-mo-old, female, OVX Wistar rats.^36^ The study reported a larger zone of apoptotic osteocytes in the osteotomies of the osteoporotic group compared to the control, suggesting that increased bone resorption and slower bone formation are consequences of an increased apoptotic response in the osteoporosis phenotype.^36^ Estrogen withdrawal has been previously shown to induce osteocyte death by apoptosis and stimulate preferential local resorption via high concentrations of apoptotic osteocytes in both rodent and human tissue samples.^37^^,^^38^ Furthermore, it has been shown that disruption of bone remodeling pathways, such as the RANKL/OPG system, and signaling pathways, including BMPs, Wnt/β-catenin, and TGF-β, ultimately result in the elevated bone resorption and reduced bone formation seen in osteoporosis.^39^ Impaired structural integrity, angiogenesis, and bone fluid transport, further diminish wound healing capabilities in osteoporotic bone.^40^ Overall, declining cellular functions decreased osteoblastic activity, reduced mesenchymal stem cell differentiation potential, and limited availability of precursor cells essential for bone formation associated with aging may have contributed to the imbalance in bone remodeling dynamics observed (Figure 6).^41^

In the current study, 6-mo-old, OVX and sham-operated animals following tooth extraction revealed a clinical appearance consistent with alveolar osteitis; no significant difference in BMD was found in the healed extraction sockets between the experimental groups. Defective healing was characterized by areas of necrotic, exposed bone, and exfoliated bony sequestrae, along with a tendency for decreased BMD in maxillary alveolar bone in the OVX group compared to the sham. A quantitative radiological assessment of the new bone formation within the alveolar socket in the present study could not be performed due to the resorption of the alveolar ridge showing a minimally detectable alveolar socket boundary. Previous preclinical data demonstrated the ability of ABL to increase BMD in standard reference sites such as the femur, tibia, and lumbar vertebrae in younger, OVX rats.^42^ This study provides the first evidence of bone density-enhancing effects of ABL on alveolar bone, as the maxillary alveolar BMD was significantly higher in the OVX-ABL group vs the sham-VEH and OVX-VEH groups. Most of the implant samples displayed histological signs of peri-implant osteonecrosis after 14 and 21 d in both treatment groups (OVX-VEH and OVX-ABL). The peri-implant defects demonstrated minimal new bone formation or a complete absence of osseous filling, accompanied by osteolysis around the implant. In this type of peri-implant defect, a defect fill of more than 20% is typically expected after 14 d in young gonad-intact male rats.^28^ Peri-implant bone fill and peri-implant BMD within the peri-implant defect did not exhibit significant changes in either group. These results contrast with multiple studies our group has published using the same implant model in younger animals showing normal tooth extraction socket healing and implant osseointegration.^26^^,^^28^^,^^43-45^

While the present study provides new information on extraction socket healing in the maxillary alveolar bone of 6-mo-old, OVX female Sprague–Dawley rats, the adverse healing outcomes represent a limitation of the study and should be considered when interpreting adverse outcomes in peri-implant healing. Furthermore, the current study only used female rats, limiting the generalizability of findings to males, and additional animal groups with varying ages or periods of bone depletion were not included. While the current model simulates osteoporosis in rats that are older than what is conventionally used for dental extraction studies, differences in bone metabolism, anatomy, and physiology between rats and humans should be considered when extrapolating the findings to human populations. Whether osteoporosis and aging have a synergistic negative impact on extraction socket healing in humans remains unknown, as patients directly undergo osteoporosis therapy and are not included untreated in clinical studies.^46^ However, it is known that trauma, infection, or inflammation can trigger osteonecrosis without antiresorptive or antiangiogenic medications in humans with osteoporosis.^47^ Specific to the aging process in rodents, radicular hypercementosis and hypermineralization of cementum tend to occur; resultant irregularities in the apical root morphology and low elastic modulus of cementum increased the difficulty of the molar extraction procedure and may have influenced socket healing in the 6-mo-old animals.^48^^,^^49^ How aging alone affects human alveolar bone healing is scarcely investigated and based on limited existing evidence, chronological age seems to not influence implant survival.^50^ The delayed extraction socket healing and altered peri-implant bone metabolism associated with aging and estrogen deficiency may have compromised dental implant placement in the present study and masked any potential effect of ABL on implant osseointegration or peri-implant regeneration. Considering the clinical outcomes following tooth extraction in the present study, earlier administration of bone anabolic therapies at the time of ovariectomy surgery may be beneficial for future research.

## Conclusion

This study demonstrated that 6-mo-old rats with sham-operation or ovariectomy exhibited impaired tooth extraction socket healing. The observed delayed wound repair, such as alveolar osteitis, decreased BMD, and impaired osseointegration underscore the demanding nature of this model. ABL exhibited a positive effect on native alveolar bone density while limitations with poor extraction socket healing masked effects on the implant outcomes.

## Supplementary Material

Supplemental_Figure_1_ziae113

## Data Availability

The data underlying this article will be shared on reasonable request to the corresponding author.
