# Supplementary material for: Healing sequelae following tooth extraction and dental implant placement in an aged, ovariectomy model
Source: JBMR Plus. 2024 Aug 31;8(10):ziae113. doi: 10.1093/jbmrpl/ziae113 (PMC11427826; doi:10.1093/jbmrpl/ziae113)
Supplement: Supplemental_Figure_1_ziae113 [file supplemental_figure_1_ziae113.docx]

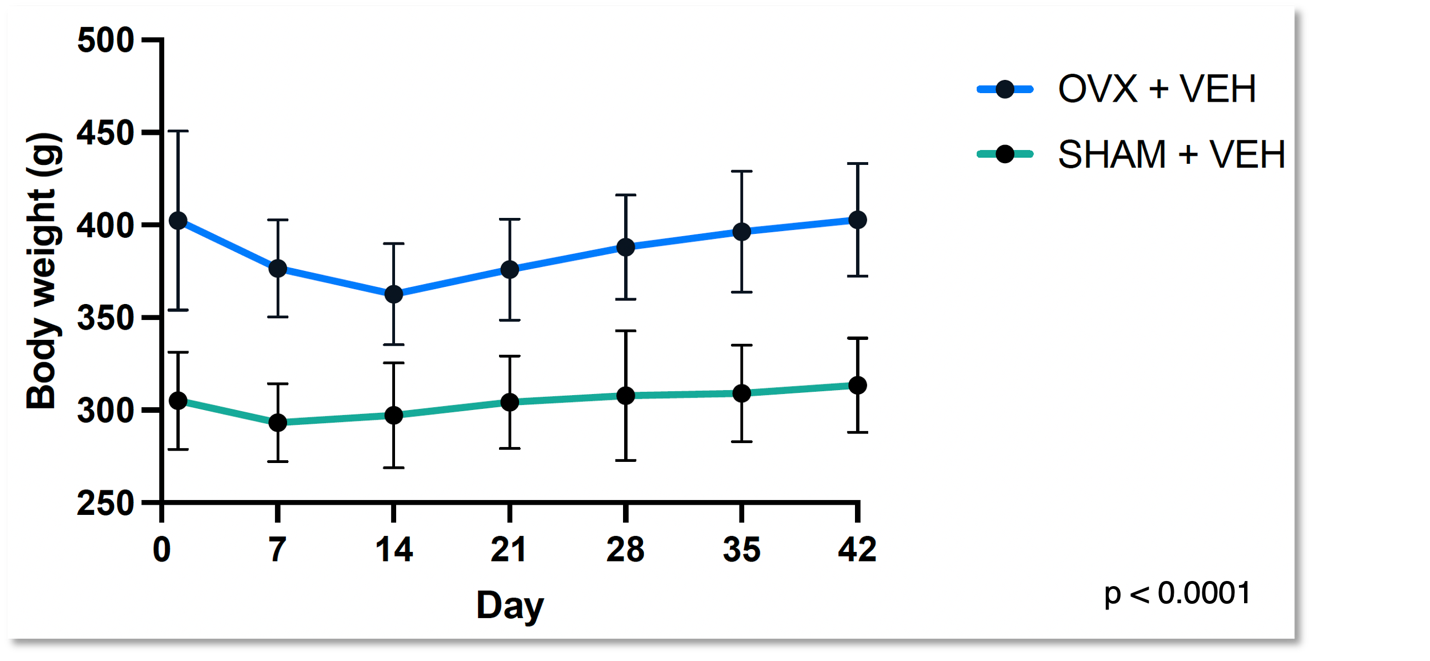


Supplemental Figure 1. Mean body weight of OVX and SHAM animals treated with vehicle injection at time of maxillary first molar extraction and 1, 2, 3, 4, 5, 6 weeks post-extraction were significantly different at all time points (p < 0.0001).
